# Supplementary material for: Glutathione Deficiency and Alterations in the Sulfur Amino Acid Homeostasis during Early Postnatal Development as Potential Triggering Factors for Schizophrenia-Like Behavior in Adult Rats
Source: Molecules. 2019 Nov 22;24(23):4253. doi: 10.3390/molecules24234253 (PMC6930621; doi:10.3390/molecules24234253)
Supplement: Supplementary file 1 [file molecules-24-04253-s001.pdf]

Glutathione deficiency and alterations in the sulfur amino acid homeostasis during early postnatal development as potential triggering factors for schizophrenia-like behavior in adult rats

Magdalena Górny[1], Agnieszka Wnuk[2], Adrianna Kamińska[3], Kinga Kamińska[2], Grażyna Chwatko[3], Anna Bilska-Wilkosz[1], Małgorzata Iciek[1], Małgorzata Kajta[2], Zofia Rogóż[2], Elżbieta Lorenc-Koci[2]

1 The Chair of Medical Biochemistry, Jagiellonian University Medical College, 7 Kopernika Street, 31-034 Kraków, Poland

2 Maj Institute of Pharmacology, Polish Academy of Sciences, 12 Smętna Street, 31-343 Kraków, Poland;

3 Department of Environmental Chemistry, University of Łódź, 163 Pomorska Street, 90-236 Łódź, Poland;

* Correspondence: lorenc@if-pan.krakow.pl

Supporting information

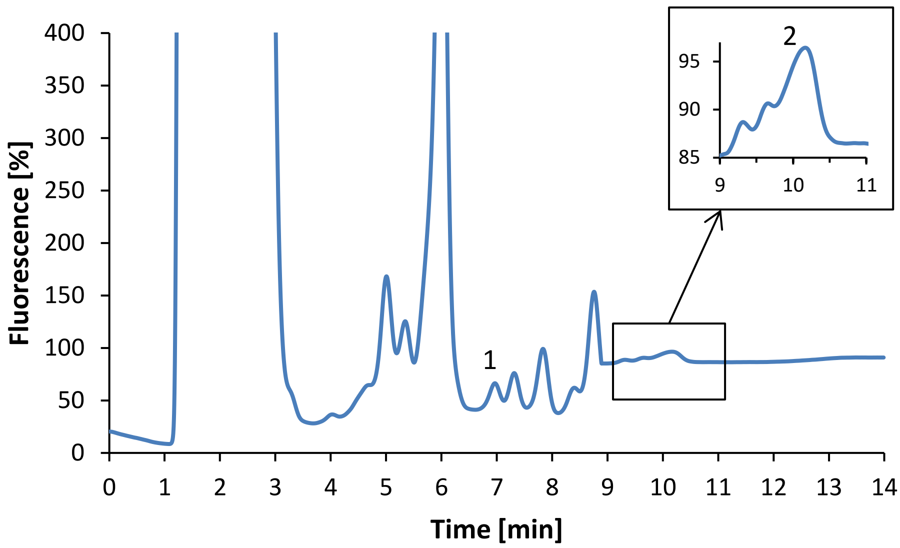

Figure S1. Representative chromatogram for methionine and homocysteine assay in the liver homogenate. Peaks: 1 - methionine, 2 - homocysteine.

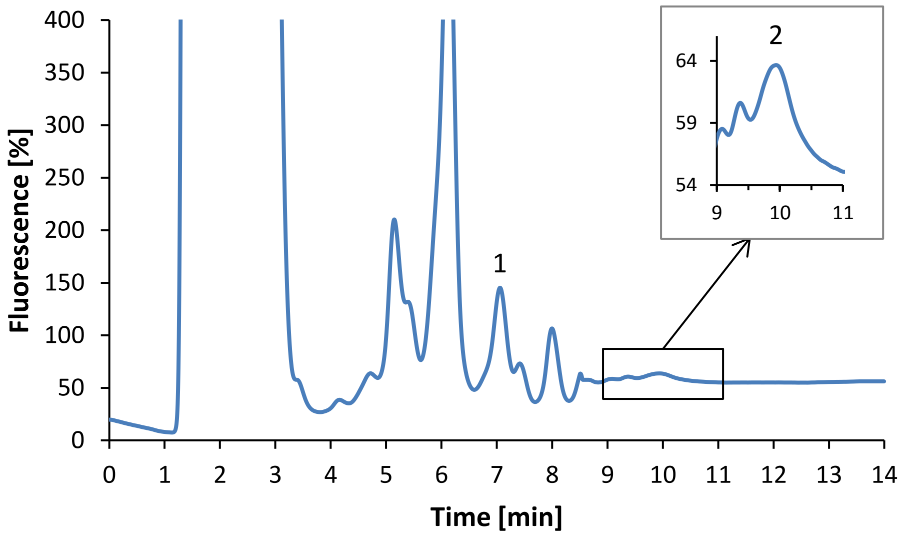

Figure S2. Representative chromatogram for methionine and homocysteine assay in the kidney homogenate. Peaks: 1 - methionine, 2 - homocysteine.

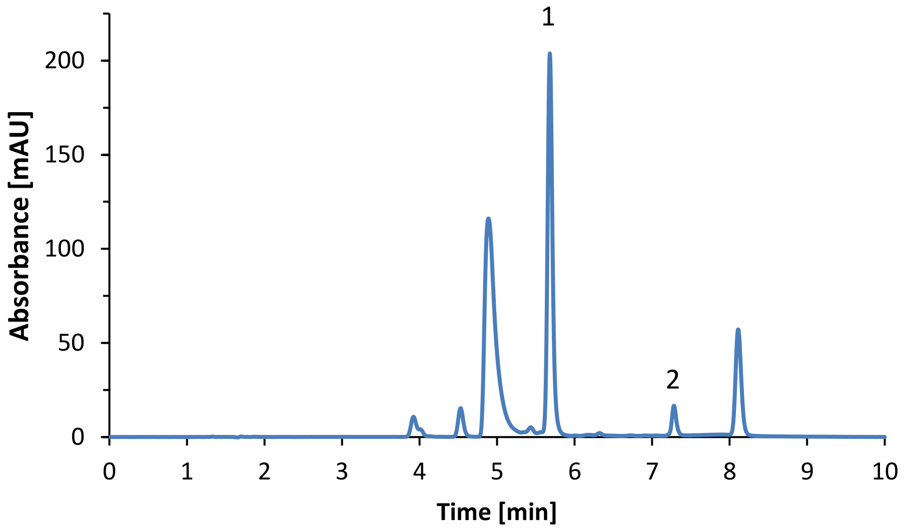

Figure S3. Representative chromatogram for glutathione and cysteine assay in the prefrontal cortex (PFC) homogenate. Peaks: 1 - glutathione, 2 - cysteine.

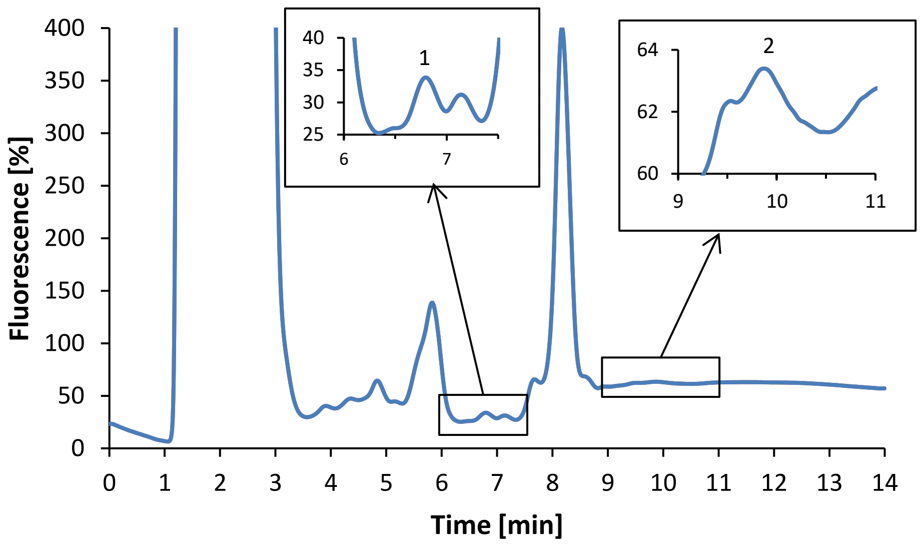

Figure S4. Representative chromatogram for methionine and homocysteine assay in the PFC homogenate. Peaks: 1 - methionine, 2 - homocysteine.

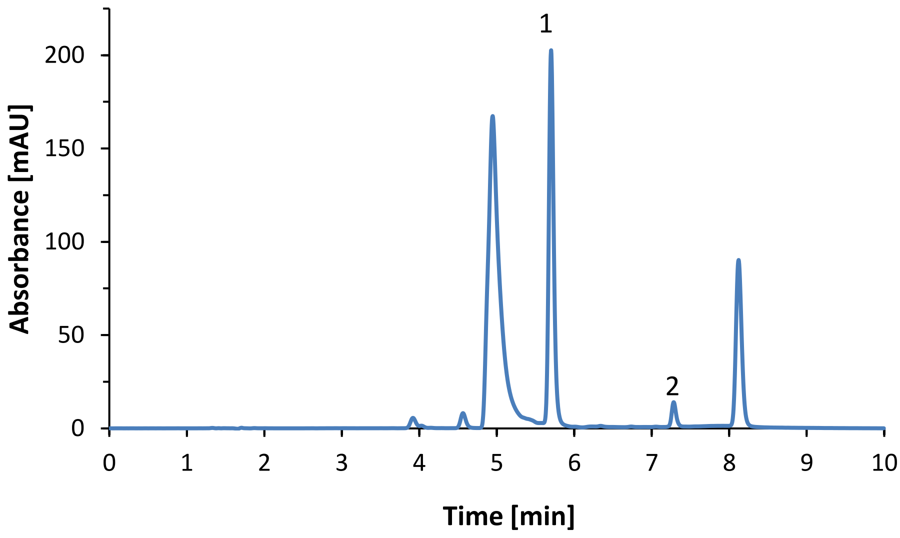

Figure S5. Representative chromatogram for glutathione and cysteine assay in the hippocampus (HIP) homogenate. Peaks: 1 - glutathione, 2 - cysteine.

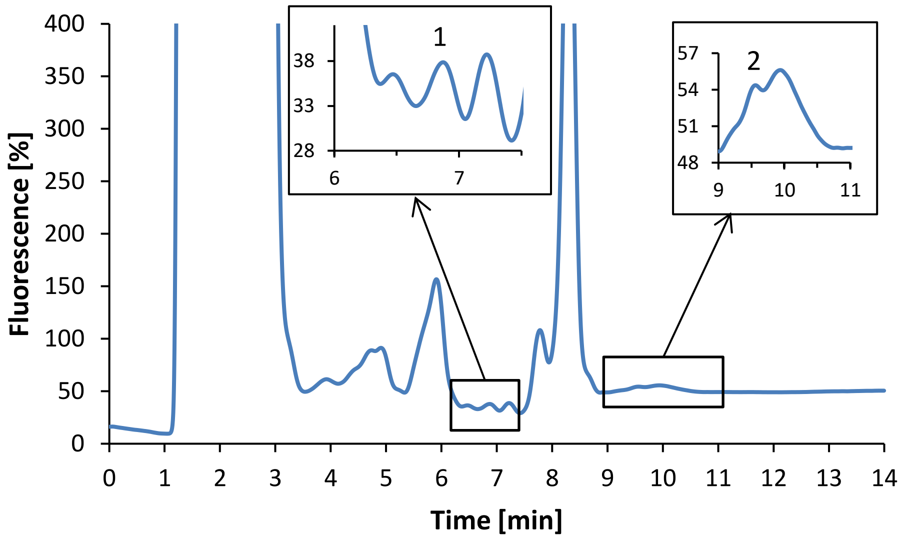

Figure S6. Representative chromatogram for methionine and homocysteine assay in the HIP homogenate. Peaks: 1 - methionine, 2 - homocysteine.
